# A qEEG-based prognostic model for cognitive impairment after ischemic stroke: Development and internal validation

**DOI:** 10.1016/j.ibneur.2026.06.021

**Published:** 2026-07-02

**Authors:** Vy Tuong Nguyen, Trang Ho Thu Quach, Hiep Do, Kien Trung Duong, Khoa Nhat Bui, Huong Ha, An Gia Pham, Thang Cong Tran

**Affiliations:** a Neurology Department, University of Medicine and Pharmacy at Ho Chi Minh City, Vietnam; b Neurology Department, Cho Ray Hospital, Ho Chi Minh City, Vietnam; c Faculty of Information Technology, Ho Chi Minh City Open University, Vietnam; d International University, Vietnam National University, Ho Chi Minh City, Vietnam

**Keywords:** Ischemic stroke, Cognitive impairment, Quantitative EEG, Delta/Alpha Ratio, Peak Alpha Frequency

## Abstract

**Background:**

Cognitive impairment is a frequent and disabling consequence of ischemic stroke, yet early prognostic tools are limited. Quantitative electroencephalography (qEEG) offers a portable, low-cost measure of post-stroke cortical dysfunction. We aimed to develop a prognostic model for six-month cognitive impairment after first-ever ischemic stroke.

**Methods:**

In this single-centre prospective cohort study, patients with first-ever supratentorial ischemic stroke (NIHSS < 15) were enrolled within 7 days of onset at a tertiary hospital in Vietnam. Resting-state 19-channel EEG and brain MRI were acquired at baseline. Four region-specific qEEG parameters were derived for five cortical regions and globally: Delta/Alpha Ratio (DAR), Delta-Theta/Alpha-Beta Ratio, Peak Alpha Frequency (PAF), and Dominant Frequency Variability. The primary outcome was cognitive impairment at six months (education-adjusted MoCA < 26). Predictors were selected by AIC-guided forward stepwise logistic regression. Internal validation used a selection-aware bootstrap (1000 replicates), with multiple imputation and sensitivity analyses. Reporting followed TRIPOD and STROBE guidelines.

**Results:**

The analytic cohort comprised 96 participants; 44 (46%) developed cognitive impairment at six months. Higher occipital DAR (OR 2.09, 95% CI 1.45–3.39), lower frontal PAF (OR 0.46, 95% CI 0.27–0.74), and education below high school (OR 7.16, 95% CI 1.89–34.18) increased risk of cognitive impairment. Infarct volume was associated univariably but did not survive multivariable selection. Apparent AUC was 0.937 (95% CI 0.890–0.984); optimism-corrected AUC was 0.891 (0.861–0.945), with good calibration (Brier score 0.098). Effects were preserved across multiple imputation and alternative MoCA cutoffs.

**Conclusions:**

In this single-centre development cohort, a parsimonious three-variable model combining occipital DAR, frontal PAF, and educational attainment showed good internal discrimination and calibration for six-month cognitive impairment after first-ever ischemic stroke. These variables should be regarded as candidate predictors rather than an established prognostic tool; external multicentre validation against clinically adjudicated endpoints is the necessary next step before any clinical interpretation.

## Highlights

Occipital DAR and frontal PAF predict 6-month post-stroke cognitive impairment.
Education below high school is an independent predictor via cognitive reserve.
Full-pipeline optimism-corrected AUC was 0.891 in a single-centre cohort.
Infarct volume was not retained when qEEG functional parameters were included.
The model is a research framework requiring external multicentre validation.

## Introduction

Cognitive impairment is a frequent and disabling consequence of ischemic stroke. It significantly affects quality of life, functional independence, and mental health. Post-ischemic stroke cognitive impairment is prevalent, reported in approximately 67% of patients within the first three months (Utomo & Pinzon, 2023), 57% at six months (Mellon et al., 2015), and 38% within the first year (El Husseini et al., 2023). The healthcare costs for stroke patients with cognitive impairment or dementia are nearly three times higher than for those without. This is largely due to the long-term care needs and reliance on informal caregivers (Hoang et al., 2020). Early detection tools for cognitive impairment in the subacute stage of stroke allow for timely intervention to optimise treatment outcomes.

Cognitive function is associated with the balance between slow and fast waves, alpha activity integrity, and brain frequency stability. Studies have linked increased delta power and reduced alpha synchronization to cognitive impairment (Fan et al., 2022, Lejko et al., 2020). Recent studies have highlighted the clinical utility of qEEG for early detection of cognitive impairment, with one study reporting that a qEEG-based index predicted post-stroke cognitive impairment with 77.4% accuracy (Schleiger et al., 2017). In patients with amnestic mild cognitive impairment (aMCI), qEEG has revealed alterations in spectral activity and neural connectivity that distinguish them from healthy controls, underscoring its potential as an early biomarker of neurodegeneration (Zawiślak-Fornagiel et al., 2024). Several qEEG parameters including Peak Alpha Frequency (PAF), Delta/Alpha Ratio (DAR), and Delta-Theta/Alpha-Beta Ratio (DTABR) have shown significant associations with cognitive performance (Schleiger et al., 2014).

The capacity of qEEG to objectively quantify neural oscillatory changes offers promise not only for early diagnosis but also for prognosis and treatment monitoring. Furthermore, its portability and cost-effectiveness make it particularly well-suited for use in routine clinical settings, especially in resource-limited environments. While qEEG provides valuable insights into brain activity, structural factors such as infarct characteristics including volume and location also play an important role in determining cognitive outcomes. Infarcts in strategic brain regions, such as the left frontotemporal lobe, left thalamus, and right parietal lobe increase the risk of cognitive impairment (Weaver et al., 2021). However, although larger infarct volumes are linked to cognitive impairment (Prodjohardjono et al., 2020), growing evidence indicates that functional disruptions reflected by qEEG are more closely associated with cognitive outcomes.

Nonetheless, existing studies have limitations, including small sample sizes (Aminov et al., 2017, Schleiger et al., 2014) and shorter post-stroke follow-up periods for cognitive assessment ranging from a few weeks (Song et al., 2015) to three months (Aminov et al., 2017). During the first few months, cognitive function is unstable and influenced by stroke itself, which may not fully reflect long-term consequences. Furthermore, in previous cross-sectional or case-control studies, these designs lack the longitudinal follow-up necessary to assess the progression of cognitive impairment over time (Hadiyoso et al., 2023, Petrovic et al., 2017). The overuse of global qEEG parameters and underuse of region-specific ones might miss important localized brain activity associated with post-stroke cognitive impairment (Aminov et al., 2017).

To address these gaps, we conducted a prospective cohort study of patients with first-ever ischemic stroke, integrating qEEG, Magnetic Resonance Imaging (MRI), clinical, and demographic variables to develop a predictive model of cognitive impairment at six months post-stroke. Our study aims to identify high-risk patients for targeted interventions to improve long-term cognitive outcomes.

## Methods

### Study Design and Participants

This prospective cohort study was conducted at Cho Ray Hospital, Ho Chi Minh City, Vietnam, one of the five largest tertiary care centres in the country, from September 2023 to August 2024. Eligible participants met the following inclusion criteria: (1) first-ever supratentorial ischemic stroke, (2) NIHSS score < 15, (3) stroke onset within the past 7 days of hospitalization, and (4) willingness to complete cognitive assessments at 6 months post-stroke. Exclusion criteria at baseline included: (1) prior diagnosis of dementia (IQCODE score ≥ 3), (2) history of brain injury, (3) major psychiatric illness (e.g., major depression, schizophrenia), and (4) severe language impairments such as aphasia or severe dysarthria.

*Handling of intercurrent events***:** Patients who experienced a recurrent ischemic stroke between baseline assessment and the six-month MoCA evaluation (n = 18) were excluded from the analytic cohort under a pre-specified intercurrent-event handling strategy, following international guidance for handling events that occur between baseline measurement and outcome assessment (ICH-E9(R1) 2019). The rationale is that the six-month MoCA in such patients reflects the combined cognitive impact of two ischemic events, whereas the baseline qEEG and MRI biomarkers characterise only the index stroke. The model is therefore explicitly developed as a prognostic tool for first-ever ischemic stroke.

Sample-size requirements were evaluated using the framework of Riley et al. (Riley et al., 2019), implemented in the pmsampsize R package, using the final-model parameter count (k = 3), an anticipated AUC of 0.85, and an anticipated outcome proportion of 0.30. Required minimum sample sizes by criterion were: shrinkage ≥ 0.9, n ≥ 81; optimism ≤ 0.05, n ≥ 71; R² difference ≤ 0.05, n ≥ 34; outcome proportion margin ± 0.05, n ≥ 323. The analytic cohort (n = 96) meets criteria (i)–(iii) prospectively. Criterion (iv) is met at the relaxed margin of ± 0.10 (n ≥ 81) but not at the default ± 0.05. This study was conducted and reported in accordance with the TRIPOD and STROBE reporting guidelines.

### Clinical data collection

Baseline data included demographic information, vascular risk factors (hypertension, diabetes, dyslipidemia, atrial fibrillation, ischemic heart disease), and stroke characteristics (NIHSS score, lesion laterality, and vascular territory). Stroke and transient ischemic attack histories were confirmed by clinical examination and MRI findings.

### Vascular territory classification

The vascular territory involved was determined according to established anatomical arterial distributions based on lesion location on DWI (Tatu et al., 1998). Infarcts were categorized into anterior cerebral artery, anterior choroidal artery, middle cerebral artery, internal carotid artery, or posterior cerebral artery territories. Classification was performed by a board-certified neurologist blinded to clinical and cognitive outcome data. In cases involving multiple territories, the predominant territory was assigned based on the largest infarct extent.

### Imaging data collection

Brain MRI was performed during the early subacute phase, between 5 and 8 days after stroke onset, prior to EEG acquisition, using 1.5 T scanners (Siemens Avanto and Siemens Aera). Diffusion-weighted imaging (DWI) sequences were used for infarct delineation. Infarct volume was quantified by manual slice-by-slice segmentation on axial DWI (5 mm slice thickness, no inter-slice gap) using MRIcro software, with infarcted regions defined as areas of DWI hyperintensity confirmed by corresponding ADC restriction. Hemorrhagic transformation, if present, was included in the total volume (Wong et al., 2022).

Two independent raters, blinded to clinical characteristics, EEG findings, and cognitive outcomes, performed lesion segmentation separately. The mean of the two raters' volumes was used as the analytic variable. Inter-rater reliability was assessed using the intraclass correlation coefficient for the average of *k* = 2 raters under a two-way random-effects, absolute-agreement model, ICC(2,k) (Koo & Li, 2016). Agreement was excellent (ICC[2,*k*] = 0.955, 95% CI 0.93–0.97), with no systematic between-rater bias (mean bias −1.26 cm³, Wilcoxon *p* = 0.20). Bland-Altman 95% limits of agreement on the log scale were 0.42–2.14 (ratio R1/R2).

White matter hyperintensities were rated on axial FLAIR images using the Fazekas scale (Fazekas et al., 1987), with periventricular and deep WMH scored separately on a 0–3 scale. Global cortical atrophy was assessed using the Scheltens visual rating scale (0–3) (Scheltens et al., 1993).

### EEG recording and preprocessing

Resting-state, eyes-closed EEG was recorded in the stroke unit predominantly on day 7 post-stroke (85.4%; range 7–9) using a Natus EEG system with 19 scalp electrodes positioned according to the international 10–20 system. Signals were digitized at 500 Hz using a linked-ears reference as configured on the Natus clinical EEG system, with electrode impedance maintained below 5 kΩ. Acquisition proceeded only when patients were afebrile, free of electrolyte imbalance, and not receiving sedative agents, benzodiazepines, antiepileptic drugs, or continuous sedation; alertness was formally assessed before and after acquisition, and wakefulness was maintained via verbal reminders at two-minute intervals. The acquisition reference was retained; no software-based re-referencing was applied. A linked-ears reference is symmetric across hemispheres, avoiding lateralized bias. Although reference choice can affect spectral estimates, relative measures such as ratios and peak frequency are less sensitive to it than absolute power (Yao et al., 2019).

**Fig. 1:**
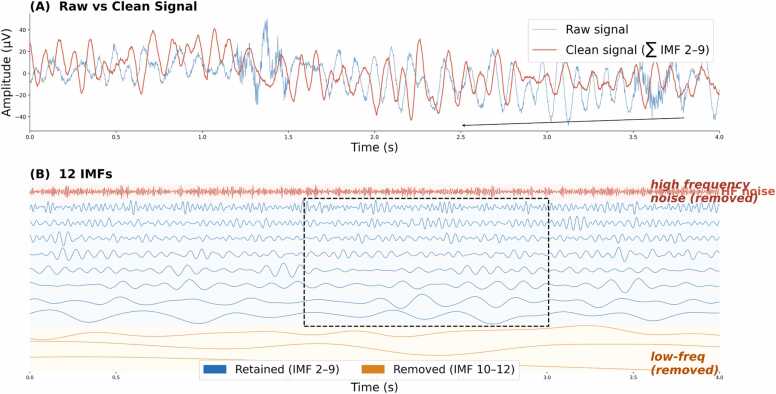
Illustration of mEMD-based EEG artifact removal (Stage 1). *Panel (A) Representative raw EEG signal (blue) overlaid with the reconstructed clean signal (red) after mEMD decomposition. (B) The 12 intrinsic mode functions (IMFs) extracted from a representative channel. IMF 1 (red) representing high-frequency noise and EMG contamination, and IMF 10–12 (orange) representing low-frequency drift and baseline wander, were excluded from signal reconstruction. The remaining IMFs 2–9 (blue) encompassing physiological frequency bands were retained and summed to yield the clean signal. The dashed box highlights the retained IMF components.*

Recordings were converted from Nicolet (.e) to EDF using FieldTrip (Oostenveld et al., 2011) and processed in Python (MNE, SciPy); non-EEG channels were excluded. Preprocessing proceeded through three sequential, algorithmically defined stages, with all artifact decisions following predefined rules applied uniformly across participants (no manual epoch rejection or amplitude thresholding). First, each channel was decomposed by masked EMD (50 Hz mask) into 12 intrinsic mode functions (IMFs) (Deering and Kaiser, 2005, Do et al., 2023); IMF 1 (high-frequency noise/EMG) and IMFs 10–12 (low-frequency drift) were discarded, and IMFs 2–9 summed to reconstruct the denoised signal (Fig. 1). Second, the reconstructed signal was segmented into consecutive non-overlapping 30-second epochs and band-pass filtered (1–40 Hz) using a zero-phase FIR filter (MNE-Python Hamming-windowed design); no notch filter was applied. Third, FastICA was applied chunk-wise to each 30-second segment (19 components per segment), with ocular components rejected by a pre-trained binary classifier (Do et al., 2022). Recordings with at least three minutes of cleaned EEG (met by all included participants) were retained for analysis.

### Quantitative EEG (qEEG) analysis

**Fig. 2:**
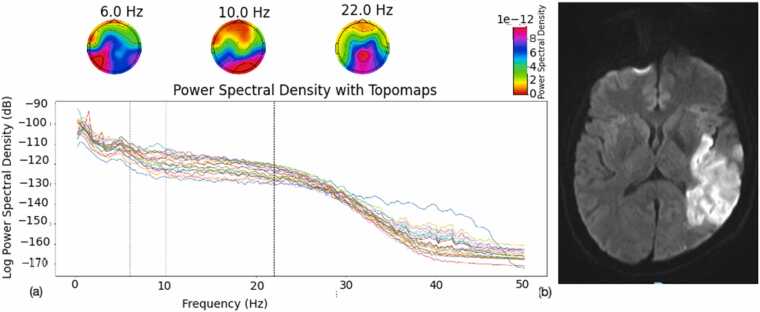
EEG power spectral density and diffusion-weighted MRI in a post-stroke patient. *Panel (a) shows the power spectral density (PSD) with corresponding topographic maps at 6.0 Hz, 10.0 Hz, and 22.0 Hz. Panel (b) presents diffusion-weighted MRI (DWI) demonstrating infarcted brain regions. The patient is a 58-year-old female with an NIHSS score of 6 at admission and a MoCA score of 6 at the 6-month follow-up*.

Power spectral density (PSD) was estimated using Welch's method (scipy.signal.welch) with 10-second segments, Hann window, no overlap, and median averaging across segments for robustness to residual transient artifacts. PSD was computed per channel and aggregated across five anatomically defined regions, with an additional global average. The five regions were frontal (Fp1, Fp2, F3, F4, F7, F8, Fz), central (C3, C4, Cz), temporal (T3, T4, T5, T6), parietal (P3, P4, Pz), and occipital (O1, O2) (Fig. 2). Frequency bands were defined as delta (1–4 Hz), theta (4–8 Hz), alpha (8–13 Hz), and beta (13–30 Hz); band power was obtained by summing PSD bins within each band's frequency range.

Based on prior literature linking oscillatory slowing and alpha rhythm disruption to post-stroke cognitive impairment, four quantitative EEG parameters were derived for each region (Schleiger et al., 2014; Schleiger et al., 2017; Zhao et al., 2025). Delta/Alpha Ratio (DAR) was calculated as delta power divided by alpha power. Delta-Theta/Alpha-Beta Ratio (DTABR) was calculated as (delta + theta) / (alpha + beta). Peak Alpha Frequency (PAF) was defined as the frequency of maximal spectral power within 5.62–12.89 Hz. Dominant Frequency Variability (DFV) was computed as the standard deviation of the dominant frequency in the 4–13 Hz range across consecutive non-overlapping 2-second epochs, with the dominant frequency in each epoch identified via FFT. All extracted features were exported for subsequent statistical modelling.

### qEEG quality review

Following parameter extraction, qEEG values across the 24-variable panel were visually inspected for physiological plausibility, and the visual identification was confirmed by four algorithmic methods (univariate z-scores, modified z-scores via MAD, classical and robust Mahalanobis distances). The exclusion criterion was applied post-hoc and was not pre-specified.

### Follow-up and cognitive assessment

Cognitive function was assessed at six months post-stroke using the Montreal Cognitive Assessment (MoCA). In accordance with standard MoCA scoring guidelines, one additional point was added for participants with ≤ 12 years of formal education. Cognitive impairment was defined as an education-adjusted MoCA score < 26. This 12-item screening instrument assesses several cognitive domains, including visuospatial/executive function, naming, attention, language, abstraction, delayed recall/memory, and orientation. Cognitive assessors were blinded to qEEG and imaging findings.

### Statistical analyses

The primary objective was to identify qEEG parameters predictive of cognitive impairment at six months in patients with first-ever ischemic stroke. Descriptive statistics were presented as frequencies and percentages for categorical variables, and medians with interquartile ranges (IQR) for non-normally distributed continuous variables. Age was analysed continuously and additionally categorised for descriptive comparisons as < 50 years (American Heart Association young-onset stroke definition), 50–64 years, and ≥ 65 years (World Health Organization criteria for older adults) (Putaala et al., 2025). Group comparisons between participants with and without cognitive impairment used Chi-square or Fisher's exact tests for categorical variables and the Mann–Whitney *U* test for continuous variables.

Predictor selection used forward stepwise logistic regression guided by the Akaike Information Criterion (AIC). Univariable screening yielded 23 candidates: 21 qEEG parameters (central, temporal, and global DFV excluded for lack of significant association) and 2 clinical variables (education level and infarct volume). Variables were entered in ascending order of univariate AIC, starting with occipital DAR, with previously entered variables re-evaluated and those losing significance removed. The final model retained only statistically significant predictors. Multicollinearity was assessed using Variance Inflation Factor (VIF; all VIFs <1.2). Linearity for occipital DAR and frontal PAF was assessed using restricted cubic splines with 3 knots, with likelihood-ratio tests confirming no significant departure from linearity (Fig. S2). Apparent discriminative performance was quantified by the area under the receiver operating characteristic curve (AUC); 95% CI was computed using DeLong's method (DeLong et al., 1988).

Internal validation used two complementary bootstrap procedures, reflecting distinct estimands. The full-pipeline (selection-aware) bootstrap (1000 replicates) estimated AUC optimism: within each replicate, univariable screening and forward stepwise selection (AIC-guided forward entry; backward removal at p ≥ 0.05) were repeated on the resampled data, and the bootstrap-fitted model was applied to the original cohort to compute test AUC (Steyerberg, 2019). Per-replicate optimism was apparent AUC minus test AUC; the optimism-corrected AUC was apparent AUC minus mean optimism, with a 95% percentile confidence interval from the test-AUC distribution. The standard (selection-naive) bootstrap (10,000 replicates) estimated coefficient shrinkage and 95% confidence intervals for the final predictors, with the predictor set held fixed; bias-corrected and accelerated (BCa) intervals are reported. Calibration was assessed using a calibration plot with 10,000 bootstrap replicates, with performance quantified by mean absolute error, mean squared error, and the Brier score.

Several sensitivity analyses assessed robustness of the final model. (1) Attrition and multiple imputation: The 23 missing six-month outcomes were handled with multiple imputation by chained equations (MICE; m = 50, missing-at-random assumption), conditional on the final-model predictors, baseline covariates that differed between analytic and excluded participants (atrial fibrillation, Fazekas score, Scheltens score, temporal PAF, dyslipidemia), and standard confounders (age, sex, NIHSS, infarct volume, hypertension, diabetes). The two outlier recordings were excluded prior to imputation, consistent with the primary analysis, as their exclusion reflects a qEEG data-quality issue independent of the outcome-missingness mechanism. The model was refitted on each imputed dataset (n = 119); and pooled by Rubin's rules (mice R package), with pooled AUC from DeLong's within-imputation variances (Marshall et al., 2009, van Buuren, 2018). To probe departures from missing-at-random, a pattern-mixture (δ-adjustment) analysis added a sensitivity parameter δ to the imputed log-odds of impairment for the lost-to-follow-up cases (δ = 0 = MAR; δ > 0 = dropouts more impaired than predicted), refitting across δ = −2 to + 5 (Table S11). (2) Outlier exclusion: The final model was refitted under five outlier-exclusion configurations to assess robustness to the post-hoc qEEG quality review. (3) Outcome adjustment and threshold choice: The model-development procedure was repeated using the unadjusted MoCA outcome and across alternative raw-MoCA cutoffs from < 22 to < 27 to assess criterion contamination and threshold robustness. (4) qEEG measurement timing: qEEG values across recording days 7–9 were compared by Kruskal–Wallis tests on all 24 qEEG variables, with Bonferroni and Benjamini–Hochberg (FDR) corrections and effect sizes quantified by epsilon-squared (ε²). The final 3-predictor model was refitted with time-from-stroke as a continuous covariate. All analyses were performed using R version 4.4.3 (R Foundation for Statistical Computing, Vienna, Austria).

### Ethical considerations

The study protocol was approved by the Institutional Ethics Committee of Cho Ray Hospital, Ho Chi Minh City, Vietnam (Approval No. 1628/GCN-HĐĐĐ, approved on September 19, 2023). The study was conducted in accordance with the Declaration of Helsinki. Written informed consent was obtained from all participants.

## Results

### Study enrolment and follow-up

**Fig. 3:**
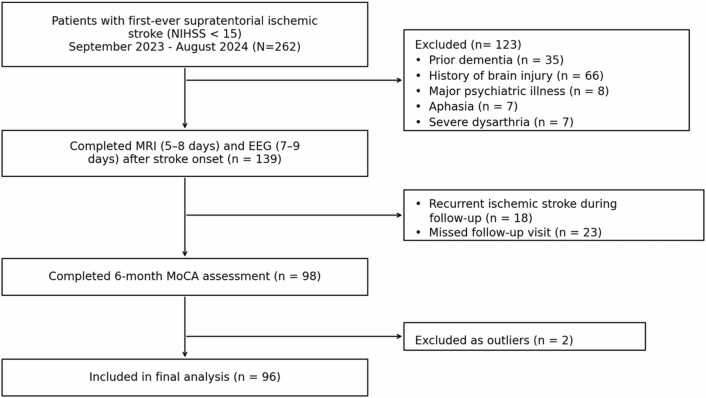
Inclusion and exclusion flow chart of study.

Of 262 patients screened for eligibility, 123 were excluded for the following reasons: history of brain injury (n = 66), prior diagnosis of dementia (n = 35), aphasia (n = 7), major psychiatric illness (n = 8), and severe dysarthria (n = 7). The remaining 139 eligible patients were enrolled with complete baseline data collection. During the six-month follow-up period, 18 participants experienced recurrent ischemic stroke and were excluded per the pre-specified design criterion, leaving 121 first-ever stroke participants event-free during follow-up. Of these, 23 were lost to follow-up before the six-month assessment, yielding 98 participants with available outcome data. Two participants were further excluded by post-hoc visual inspection for physiologically implausible values across the 24-variable qEEG panel (maximum z-scores +9.3 and +6.1). The final analytic sample comprised 96 patients with 44 events of cognitive impairment (MoCA < 26 at 6 months) (Fig. 3).

### Baseline characteristics and MoCA scores

The median age of patients was 62 years old. Around two-thirds of the participants were male, and 42% completed high school or higher. Most of the participants had hypertension (78%) and dyslipidemia (97%). The median NIHSS score was 6 (IQR 3–7). The incidence of cognitive impairment was 46% at 6 months. Patients with MoCA less than 26 were similar to patients with MoCA equal to 26 or more with regard to age, sex, hypertension, diabetes, dyslipidemia, ischemic heart disease, and NIHSS score at enrolment, though they differed by education. Patients with MoCA less than 26 were more likely to have an education level lower than high school, compared to patients with MoCA equal to 26 or more (82% vs. 39%) (Table 1).

**Table 1:** Characteristics of 96 patients, stratified by MoCA at 6 months post-stroke.

Variables	Overall (n = 96), n (%)	MoCA score at 6 months		p-value
		Less than 26 (n = 44), n (%)	26 or more (n = 52), n (%)	
Age (years), median (IQR)	62 (55 – 70)	65 (55 – 70)	61 (55 – 67)	0.160
Age category				
< 50	11 (11.5)	3 (6.8)	8 (15.4)	0.121
50 – 64	41 (42.7)	16 (36.4)	25 (48.1)	
≥ 65	44 (45.8)	25 (56.8)	19 (36.5)	
Sex, male	62 (64.6)	31 (70.5)	31 (59.6)	0.372
Education level				
< high school	56 (58.3)	36 (81.8)	20 (38.5)	< 0.001
Vascular risk factors				
Hypertension	75 (78.1)	34 (77.3)	41 (78.8)	1.000
Diabetes	36 (37.5)	18 (40.9)	18 (34.6)	0.672
Dyslipidemia	93 (96.9)	44 (100.0)	49 (94.2)	0.303
Atrial fibrillation	3 (3.1)	1 (2.3)	2 (3.8)	1.000
Ischemic heart disease	18 (18.8)	12 (27.3)	6 (11.5)	0.088
NIHSS, median (IQR)	6 (3 – 7)	6 (4 – 8)	5 (3 – 7)	0.092

IQR: Interquartile Range, NIHSS: National Institute of Health Stroke Scale.

### MRI markers and MoCA scores

The proportion of patients with affected arteries was 74% for middle cerebral artery, 13% for posterior cerebral artery, and 9% for anterior cerebral artery. Almost 95% of participants had Scheltens score of 0 (no atrophy) or 1 (mild atrophy) while around 70% of patients had a Fazekas score of 0 or 1. Patients with MoCA less than 26 were similar to patients with MoCA equal to 26 or more in all MRI characteristics, except for infarct volume. Patients with MoCA less than 26 were more likely to have greater infarct volume than patients with MoCA equal to 26 or more (median 4.50 vs. 1.33 cm^3^) (Table 2).

**Table 2:** MRI characteristics for 96 patients, stratified by MoCA at 6 months.

Variables	Overall (n = 96), n (%)	MoCA at 6 months post-stroke		p-value
		Less than 26 (n = 44), n (%)	26 or greater (n = 52), n (%)	
Affected arteries				
Anterior cerebral artery	9 (9.4)	7 (15.9)	2 (3.8)	0.075
Anterior choroidal artery	4 (4.2)	3 (6.8)	1 (1.9)	0.330
Internal carotid artery	3 (3.1)	3 (6.8)	0 (0.0)	0.093
Middle cerebral artery	71 (74.0)	31 (71.0)	40 (76.9)	0.627
Posterior cerebral artery	12 (12.5)	3 (6.8)	9 (17.3)	0.214
Infarct volume in cm^3^, median IQR	2.44 (1.02 – 8.75)	4.50 (1.46 – 30.53)	1.33 (0.80 – 3.70)	< 0.001
Scheltens score				
0	58 (60.4)	23 (52.3)	35 (67.3)	0.170
1	33 (34.4)	17 (38.6)	16 (30.8)	
2 or 3	5 (5.2)	4 (9.1)	1 (1.9)	
Fazekas score				
0	35 (36.5)	11 (25.0)	24 (46.2)	0.100
1	32 (33.3)	17 (38.6)	15 (28.8)	
2 or 3	29 (30.2)	16 (36.4)	13 (25.0)	
Hemisphere with infarct				
Dominant hemisphere	42 (43.8)	21 (47.7)	21 (40.4)	0.539
Nondominant hemisphere	52 (54.2)	22 (50.0)	30 (57.7)	
Both	2 (2.1)	1 (2.3)	1 (1.9)	

### qEEG measures and MoCA scores

**Fig. 4:**
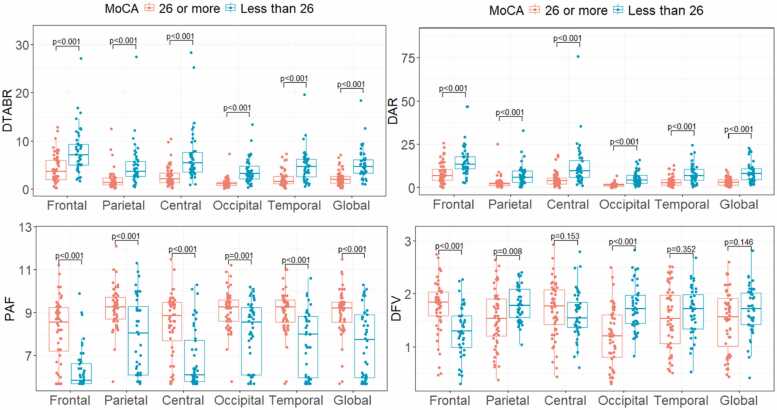
qEEG characteristics of the four parameters (DAR, DTABR, PAF, DFV) across six brain regions in the two groups: MoCA < 26 and MoCA ≥ 26.

qEEG measures were different between the MoCA < 26 and MoCA ≥ 26 groups across all six brain regions. DAR and DTABR were significantly higher in the MoCA < 26 group across all regions. Conversely, PAF was significantly lower in the MoCA < 26 group across all regions, particularly in the frontal region, indicating disrupted neural oscillatory dynamics associated with cognitive decline. DFV was significantly lower in the frontal, parietal, and occipital regions among patients with MoCA less than 26 but showed no significant difference in the central and temporal regions (Fig. 4).

### Predictive model for cognitive impairment at 6 months

**Fig. 5:**
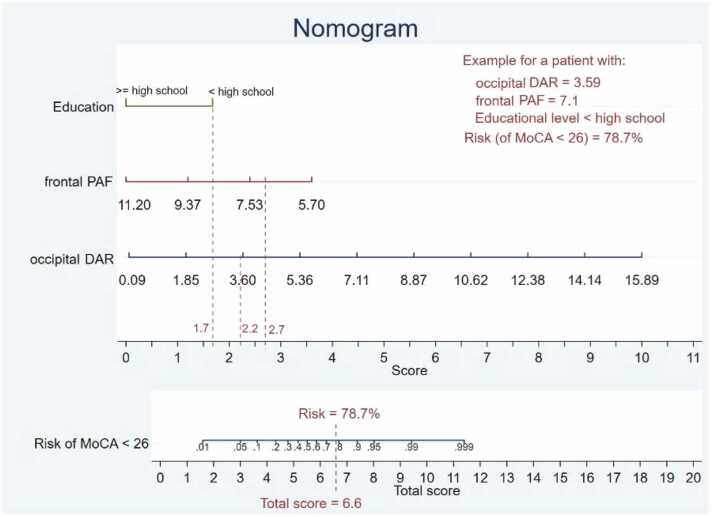
Nomogram of the final predictive model. *Based on the values of the parameters frontal PAF, occipital DAR and educational level, a score can be determined for each of these parameters as well as a total score. The corresponding risk reference for the total score can then be used to determine the risk of cognitive impairment*.

In the final multivariable logistic regression model (Table 3), higher occipital DAR (OR 2.09, 95% CI 1.45–3.39), lower frontal PAF (OR 0.46, 0.27–0.74), and education below high school (OR 7.16, 1.89–34.18) were associated with increased risk of cognitive impairment. The fitted model can be written as

$$\mathrm{logit}\left(P\right)=2.1405+0.7382\times \mathrm{Occipital}\;\mathrm{DAR}-0.7683\times \mathrm{Frontal}\;\mathrm{PAF}+1.9691\times \mathrm{Education}\_\mathrm{low}$$

$$P\left(\mathrm{cognitive}\;\mathrm{impairment}\right)=\frac{1}{1+\exp(-\mathrm{logit}\left(P\right))}$$

where occipital DAR and frontal PAF are entered on their original continuous scales and Education_low = 1 if education < high school and 0 otherwise; worked examples are provided in Supplementary Table S12. The model is presented as a nomogram for estimating individual risk in Fig. 5. The apparent area under the curve was 0.937 (95% CI 0.890–0.984), and bootstrap internal validation with full-pipeline variable selection yielded an optimism-corrected AUC of 0.891 with mean optimism of 0.046 (Fig. S3). Bootstrap-corrected odds ratios were attenuated by optimism correction (Table 3) but retained the same direction and clinical interpretation. Beyond discrimination, calibration was good (mean absolute error 0.012, mean squared error 0.00027, Brier score 0.098), with the optimism-corrected calibration curve closely following the line of identity and absolute prediction error below 3.1% for 90% of predictions (Fig. S1).

**Table 3:** Final multivariable logistic regression model for cognitive impairment (MoCA < 26 at 6 months), with bootstrap optimism correction.

Variables	Multiple logistic regression			Bootstrap Optimism-correction
	β coefficient	SE	Adjusted OR (95% CI)	Adjusted OR (95% CI)
Intercept	2.1405	1.8337	-	-
Occipital DAR	0.7382	0.2148	2.09 (1.45, 3.39)	1.70 (1.46, 2.56)
Frontal PAF	−0.7683	0.2510	0.46 (0.27, 0.74)	0.55 (0.36, 0.75)
Education < high school	1.9691	0.7245	7.16 (1.89, 34.18)	4.87 (2.13, 15.35)
AUC	-	-	0.937 (0.890, 0.984)	0.891 (0.861, 0.945)

OR: odds ratio; CI: confidence interval; AUC: area under the receiver-operating-characteristic curve. β coefficients are on the log-odds scale. The apparent AUC (95% CI) was computed by DeLong's method; the bootstrap-corrected AUC uses the full-pipeline selection-aware bootstrap (1000 replicates) in which the entire variable-selection pipeline was repeated within each resample. OR shrinkage uses standard bootstrap of the final-model coefficients.

### Sensitivity analyses

#### Attrition and multiple imputation

Baseline characteristics were compared between the 96 participants in the analytic cohort and the 25 not included (Tables S1–S3). The two groups were broadly comparable across demographic, clinical, and qEEG characteristics, with exceptions: analytic participants had higher dyslipidemia (96.9% vs 84.0%), lower atrial fibrillation (3.1% vs 16.0%), greater white matter burden (Fazekas score), and higher cerebral atrophy (Scheltens score); among qEEG parameters, only temporal PAF differed significantly. None of these differences involved the model's final predictors. Multiple imputation for the 23 participants with missing six-month outcomes produced pooled estimates closely consistent with the complete-case analysis (Table S4). This robustness held under a pattern-mixture (MNAR) sensitivity analysis: both qEEG predictors kept 95% CIs excluding 1 across δ = −2 to + 5, with only the education effect sensitive beyond δ ≈ + 1.4 (Table S11).

#### Outlier exclusion

Sensitivity analyses confirmed robustness to outlier exclusion (Table S6): frontal PAF, education, and AUC were stable across five exclusion configurations. The occipital DAR effect was sensitive to one extreme outlier (Fig. S4). The outlier's maximum z-score of + 9.3 is physiologically implausible, supporting its exclusion.

#### Outcome adjustment and threshold choice

The + 1 education-MoCA adjustment altered impairment classification of only 3 of 96 participants (3.1%). Refitting with the unadjusted outcome retained all three predictors and strengthened the education effect (aOR 9.67 vs 7.16; Table S7). Predictor effects were preserved across raw-MoCA cutoffs from < 22 to < 27 (AUCs 0.92–0.95; Table S8), confirming robustness to both threshold choice and criterion contamination.

#### qEEG measurement timing

qEEG values were stable across the 7–9 day recording window: no Kruskal–Wallis test was significant before or after Bonferroni or FDR correction, and all ε² values were below 0.05, indicating negligible effect of recording day (Table S9). Adding time-from-stroke as a continuous fourth covariate produced essentially unchanged predictor estimates and discrimination, with the time covariate itself not significant (Table S10).

## Discussion

In this prospective cohort of patients with first-ever supratentorial ischemic stroke, baseline higher occipital DAR, lower frontal PAF, and education below high school increased the risk of cognitive impairment at six months. The model demonstrated high apparent discrimination that remained good after full-pipeline bootstrap optimism correction, with well-calibrated probability estimates. Notably, although infarct volume was associated with cognitive impairment on univariable comparison, it was not retained in the multivariable model once the two regional qEEG parameters were included. These findings suggest that, in patients with mild-to-moderate first-ever ischemic stroke, the functional signature of cortical dysfunction captured by resting-state qEEG provides prognostic information that complements rather than overlaps with the structural burden of the index infarct.

The two retained qEEG predictors capture complementary aspects of post-stroke neural dysfunction. Occipital DAR reflects the magnitude of slow-wave dominance in posterior cortical regions, whereas frontal PAF reflects the temporal organisation of the residual alpha rhythm in anterior regions. The two parameters were not collinear and both retained independent significance, indicating that they carry non-redundant prognostic information about distinct dimensions of post-stroke cortical dysfunction. The occipital localisation of the slow-wave signal merits particular attention. Most index infarcts in this cohort involved the middle cerebral artery territory, with only a minority affecting the posterior circulation. The dissociation between predominantly anterior-circulation lesions and a posterior qEEG signal suggests that the predictive information arises not from local slow-wave activity at the infarct site, but from functional disturbance of posterior alpha-generating networks downstream of the lesion. This pattern is consistent with diaschisis and disruption of thalamocortical projections to posterior cortex (Babiloni et al., 2011).

Lower frontal PAF emerged as a risk factor, consistent with the role of alpha oscillations in cortical arousal and the temporal coordination of executive networks (E. Angelakis et al., 2004). Reduced PAF has been repeatedly associated with cognitive impairment across aging and dementia (Fan et al., 2022, Lejko et al., 2020), and a recent post-stroke cohort specifically reported lower PAF in patients with cognitive impairment compared with cognitively preserved stroke patients (Zhao et al., 2025). At the circuit level, the thalamus paces alpha oscillations via thalamocortical feedback loops, and cerebrovascular injury may disrupt these regulatory circuits in ways that extend beyond the lesion territory. The frontal predominance of the PAF effect in this cohort, despite the diversity of lesion locations, is consistent with the disproportionate involvement of frontal executive networks in the cognitive domains most commonly affected after ischemic stroke.

In this cohort, infarct volume did not survive multivariable selection despite a univariable association with MoCA < 26. Several explanations are plausible. First, the cohort was restricted to mild-to-moderate first-ever supratentorial ischemic stroke (NIHSS < 15), where the dynamic range of infarct volume is comparatively narrow and where strategic location may matter more than absolute volume (Weaver et al., 2021). Second, qEEG integrates the functional consequences of the infarct together with effects of pre-existing small-vessel disease, baseline network reserve, and remote (diaschisis) effects, information that a single volumetric measurement cannot capture. Third, growing evidence indicates that downstream network disruption, rather than lesion size per se, is the proximal mechanism of post-stroke cognitive impairment (Liang et al., 2024, Prodjohardjono et al., 2020, Xu et al., 2025). qEEG and infarct volume are therefore best viewed as complementary measures, each capturing different aspects of post-stroke brain status: two patients with similar infarct volumes may follow different cognitive trajectories depending on the degree of remote network dysfunction their lesion produces, and qEEG may help discriminate between them. Taken together, the present results are consistent with a network-level view of post-stroke cognitive impairment in mild-to-moderate first-ever stroke, in which qEEG serves as a more direct surrogate of the functional pathology relevant to cognition.

Education below high school was associated with a markedly increased risk of cognitive impairment, consistent with meta-analytic and cohort evidence linking higher educational attainment to better post-stroke cognitive outcomes (Contador et al., 2023, Suda et al., 2020). Education is the most commonly used proxy for cognitive reserve, which is thought to confer resilience against age- and disease-related brain changes through more efficient or flexible recruitment of neural networks (Stern, 2009). The effect was strengthened, not diminished, when the unadjusted MoCA was used. The observed magnitude is nonetheless likely inflated by unmeasured correlates of low educational attainment in this Vietnamese cohort, such as rural residence, manual occupation, and lifetime vascular exposure. The effect should be expected to attenuate in cohorts where these covariates are independently assessed. Although education itself is not modifiable, lower attainment may identify a higher-risk subgroup for prioritised cognitive surveillance and individualised rehabilitation planning.

The prospective design with prespecified six-month follow-up reduces selection and recall bias inherent in retrospective qEEG studies. Imaging raters, cognitive assessors, and the vascular-territory adjudicator were blinded to all other data, minimising information leakage between predictor and outcome ascertainment. Internal validation used a selection-aware bootstrap, complemented by multiple imputation for the 23 participants lost to follow-up, with pooled estimates consistent with the complete-case results. Sensitivity analyses across alternative MoCA cutoffs and outlier configurations preserved the direction and significance of the three predictors.

Several limitations should be acknowledged. First, this is a single-centre development cohort, and although internal validation by bootstrap provides optimism-corrected estimates, external validation in an independent multicentre population is essential before the model can be considered for clinical deployment. Second, cognitive outcome was defined by MoCA < 26 rather than by formal neuropsychological adjudication; MoCA is a sensitive screen but may classify some individuals with mild deficits as impaired without functional consequence. External validation against clinically adjudicated endpoints, including domain-specific neuropsychological batteries and functional cognitive outcomes, will be needed to confirm clinical relevance. Third, the analytic cohort was restricted to patients with NIHSS < 15 and without severe aphasia or dysarthria, so the model's performance in patients with more severe stroke or major language impairment is unknown. Fourth, 19 of 23 univariably screened qEEG candidates were not retained in the final model; while this is appropriate for a parsimonious model in a development cohort, it also implies that the precise predictors retained could change in independent samples, and external validation should evaluate the stability of variable selection as well as the model coefficients. Fifth, qEEG was acquired predominantly on day 7 (range 7–9), and although timing within this window did not measurably influence parameter values or model estimates in sensitivity analyses, the optimal timing of qEEG acquisition for prognostic purposes remains to be established. Sixth, the dataset originates from a Vietnamese tertiary care population with high rates of dyslipidemia and a particular educational distribution; generalisability to populations with different cardiovascular risk and educational profiles requires confirmation. Finally, while the model includes one structural imaging variable (infarct volume) and rich electrophysiological information, it does not incorporate strategic-lesion location, lacunar burden beyond Fazekas scoring, vascular reserve, or fluid/blood biomarkers; future iterations integrating these features may further improve prediction.

If externally validated, the three-variable model offers a parsimonious, low-cost tool for identifying patients at high risk of six-month cognitive impairment within the first week after a first-ever mild-to-moderate ischemic stroke. All three inputs (occipital DAR, frontal PAF, and educational attainment) are obtainable in a single inpatient encounter using a 19-channel clinical EEG system and a brief structured interview. The portability and cost profile of qEEG make this approach particularly suitable for resource-limited stroke services, where access to repeated neuropsychological assessment or advanced imaging is often constrained. Acquisition and analysis impose different requirements but neither undermines feasibility: acquisition uses a standard, low-cost 19-channel clinical EEG system with no added bedside burden, while the parameter-extraction pipeline runs offline and only once, requiring no specialised expertise at the point of care and packaged as shareable software. The pipeline's sophistication therefore lies in one-time algorithm development, not in any recurring cost or complexity at deployment; external validation should nonetheless test whether the predictors are reproducible under standard clinical EEG processing. A nomogram is provided to support clinical estimation of individual risk. Beyond risk stratification, the model identifies patients who may benefit from early enrolment in cognitive rehabilitation, vascular risk-factor optimisation, and structured cognitive follow-up (Xuefang et al., 2021).

External validation in independent, multicentre cohorts is the immediate priority, ideally against clinically adjudicated cognitive endpoints and domain-specific neuropsychological batteries rather than MoCA alone, to confirm both discriminative performance and the stability of the three retained predictors. Validation should extend the model to populations excluded here, including severe ischemic stroke (NIHSS ≥ 15), posterior-circulation infarction, recurrent stroke (in which a post-hoc combined-cohort check preserved the qEEG effects; Table S13), and aphasia or severe dysarthria assessed through language-independent instruments, as well as broader geographic and demographic settings. Beyond validation, qEEG-based risk stratification merits evaluation for trial enrichment, and whether qEEG-guided early intervention can modify post-stroke cognitive trajectories is a clinically important question that this model now makes testable.

Future work should externally validate this model in independent multicentre cohorts encompassing severe ischemic stroke (NIHSS ≥ 15), patients with aphasia or severe dysarthria assessed through language-independent cognitive instruments, and broader geographic and demographic settings; evaluate performance against clinically adjudicated cognitive endpoints; examine its utility for trial enrichment; and investigate whether qEEG-guided early intervention can modify the trajectory of post-stroke cognitive decline.

## Conclusions

In this single-centre development cohort of patients with first-ever supratentorial ischemic stroke and no recurrent stroke during follow-up, occipital DAR, frontal PAF, and education below high school showed good internal discrimination and calibration for cognitive impairment at six months. These variables should be regarded as candidate predictors rather than an established prognostic tool, and the apparent independence of the qEEG parameters from infarct volume is a hypothesis to be confirmed rather than a settled finding. External multicentre validation against clinically adjudicated endpoints is the necessary next step before region-specific resting-state qEEG can be considered for early prognostication of post-stroke cognitive impairment.

## CRediT authorship contribution statement

**Khoa Nhat Bui:** Visualization, Software, Formal analysis. **Huong Ha:** Writing – review & editing, Visualization, Supervision, Software, Formal analysis, Data curation. **Hiep Do:** Visualization, Software, Formal analysis. **Kien Trung Duong:** Visualization, Software, Formal analysis. **nguyen vy tuong:** Writing – review & editing, Writing – original draft, Visualization, Validation, Software, Resources, Project administration, Methodology, Investigation, Formal analysis, Data curation, Conceptualization. **Trang Ho Thu Quach:** Writing – review & editing, Writing – original draft, Validation, Formal analysis. **Thang Cong Tran:** Writing – review & editing, Writing – original draft, Resources, Project administration, Methodology, Investigation, Formal analysis, Data curation, Conceptualization. **An Gia Pham:** Writing – original draft, Visualization, Validation, Data curation.

## Funding

This research did not receive any specific grants from funding agencies in the public, commercial, or not-for-profit sectors.

## Declaration of Competing Interest

The authors declare that they have no known competing financial interests or personal relationships that could have appeared to influence the work reported in this paper.

## Footnotes

Appendix A Supplementary data associated with this article can be found in the online version at doi:10.1016/j.ibneur.2026.06.021.
