# Supplementary material for: A qEEG-based prognostic model for cognitive impairment after ischemic stroke: Development and internal validation
Source: IBRO Neurosci Rep. 2026 Jul 2;21:228–37. doi: 10.1016/j.ibneur.2026.06.021 (PMC13355503; doi:10.1016/j.ibneur.2026.06.021)
Supplement: Supplementary file 1 — Supplementary material [file mmc1.docx]

| 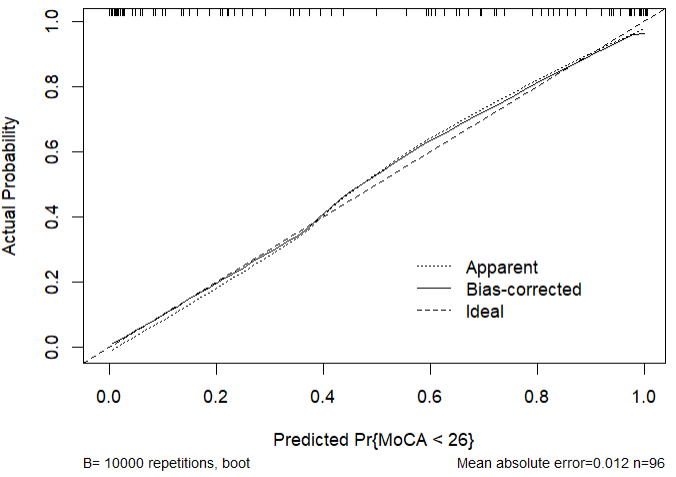 |
| --- |
| Figure S1. Bias-corrected calibration curve of the multivariable logistic regression model for predicting cognitive impairment at six months post-stroke. The apparent curve (dotted line) represents unadjusted model predictions, the bias-corrected curve (solid line) reflects optimism-adjusted predictions based on 10,000 bootstrap repetitions, and the ideal curve (dashed line) represents perfect calibration. |

| 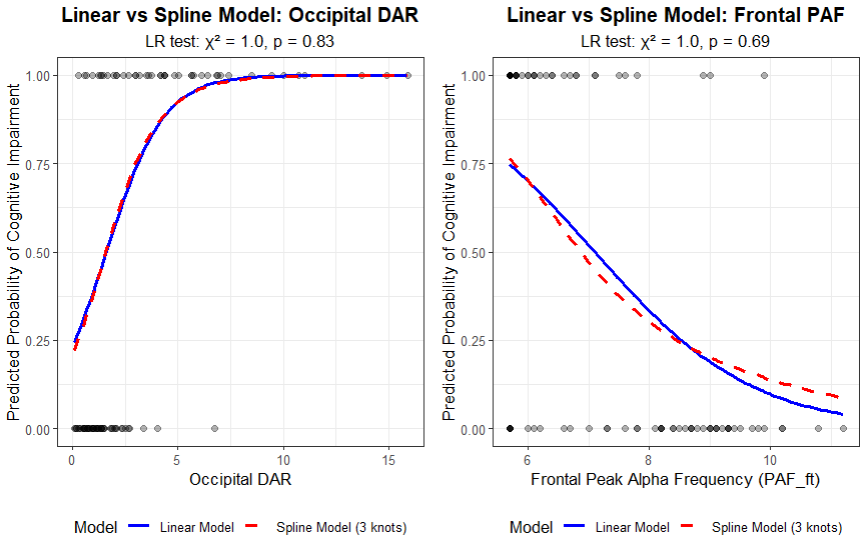 |
| --- |
| Figure S2. Linear vs. spline model comparison for continuous qEEG predictors. Left panel shows occipital DAR; right panel shows frontal peak alpha frequency (PAF). Blue solid lines represent linear models; red dashed lines represent restricted cubic spline models with 3 knots. Gray shaded areas indicate 95% confidence intervals. Rug plots at the bottom show the distribution of observations. Likelihood ratio test results are displayed in each panel header. |


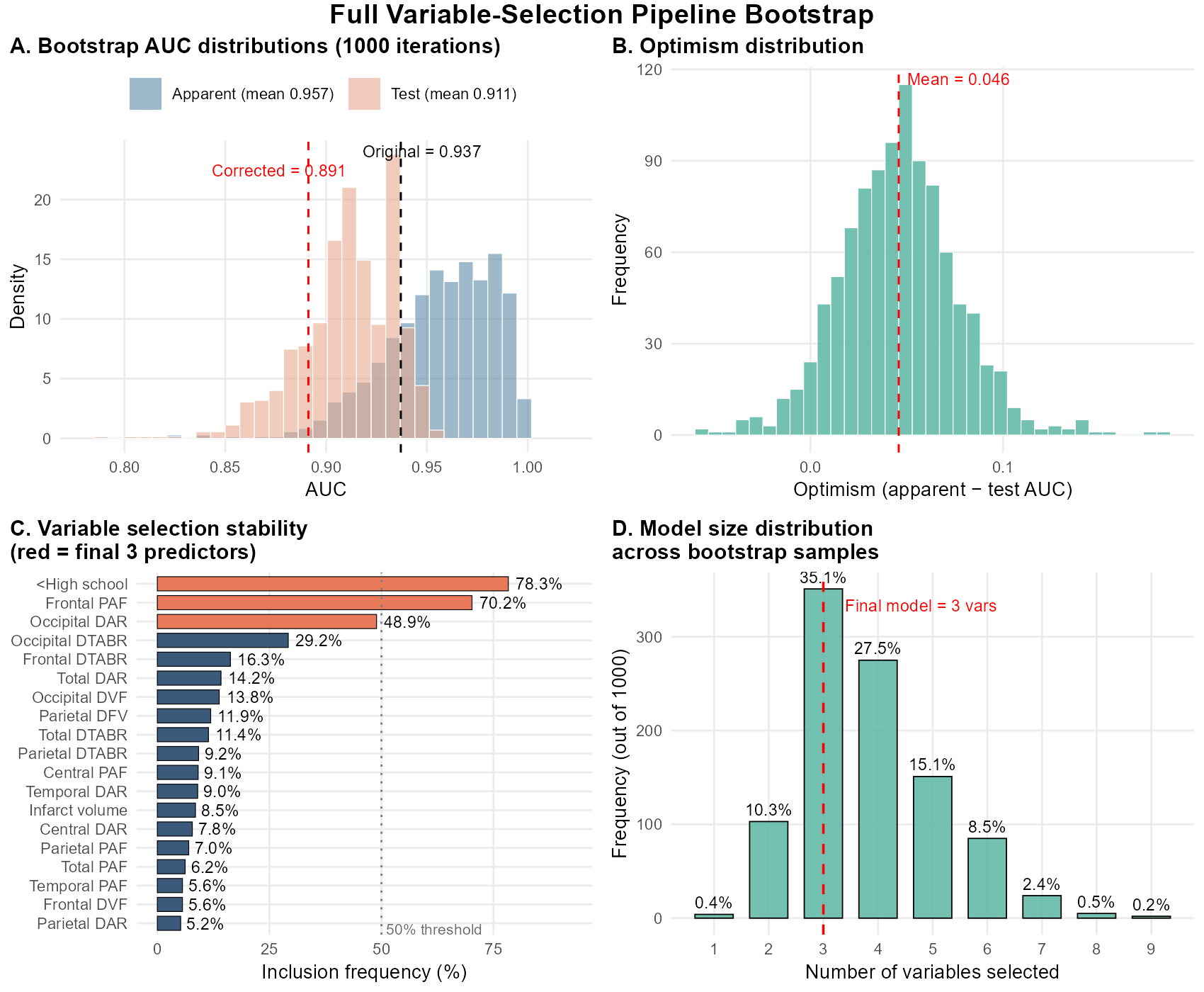


Figure S3. Full-pipeline bootstrap optimism correction (B = 1,000 replicates). (A) Distributions of apparent and test AUCs across replicates. (B) Distribution of per-replicate optimism (apparent − test). (C) Variable-level selection frequencies. (D) Distribution of model size (number of predictors retained) across replicates.

**Sensitivity analysis 1: Attrition and multiple imputation**

| Table S1. Baseline demographic and clinical characteristics of 121 enrolled participants, stratified by analytic inclusion status | | | | |
| --- | --- | --- | --- | --- |
| Variables | Overall (n=121), n (%) | Analytic inclusion status | | p-value |
|  |  | Yes (n=96),  n (%) | No (n=25),  n (%) |  |
| Age (years), median (IQR) | 63 (55 – 70) | 62 (55 – 70) | 66 (58 – 70) | 0.385 |
| Age category |  |  |  |  |
| <50 | 13 (10.7) | 11 (11.5) | 2 (8.0) | 0.501 |
| 50 – 64 | 49 (40.5) | 41 (42.7) | 8 (32.0) |  |
| ≥ 65 | 59 (48.8) | 44 (45.8) | 15 (60.0) |  |
| Sex, male | 76 (62.8) | 62 (64.6) | 14 (56.0) | 0.489 |
| Education level |  |  |  | 0.070 |
| < high school | 65 (53.7) | 56 (58.3) | 9 (36.0) |  |
| Vascular risk factors |  |  |  |  |
| Hypertension | 95 (78.5) | 75 (78.1) | 20 (80.0) | 1.000 |
| Diabetes | 45 (37.2) | 36 (37.5) | 9 (36.0) | 1.000 |
| Dyslipidemia | 114 (94.2) | 93 (96.9) | 21 (84.0) | 0.033 |
| Atrial fibrillation | 7 (5.8) | 3 (3.1) | 4 (16.0) | 0.033 |
| Ischemic heart disease | 25 (20.7) | 18 (18.8) | 7 (28.0) | 0.405 |
| NIHSS, median (IQR) | 6 (3 – 8) | 6 (3 – 7) | 5 (3 – 8) | 0.760 |

| Table S2. MRI characteristics for 121 enrolled patients, stratified by analytic inclusion status | | | | |  |
| --- | --- | --- | --- | --- | --- |
| Variables | Overall (n=121),  n (%) | Analytic inclusion status | | p-value | |
|  |  | Yes (n=96), n (%) | No (n=25), n (%) |  |  |
| Affected arteries |  |  |  |  | |
| Anterior cerebral artery | 11 (9.1) | 9 (9.4) | 2 (8.0) | 1.000 | |
| Anterior choroidal artery | 4 (3.3) | 4 (4.2) | 0 (0) | 0.580 | |
| Internal carotid artery | 3 (2.5) | 3 (3.1) | 0 (0) | 1.000 | |
| Middle cerebral artery | 94 (77.7) | 71 (74.0) | 23 (92.0) | 0.062 | |
| Posterior cerebral artery | 12 (9.9) | 12 (12.5) | 0 (0) | 0.070 | |
| Infarct volume in cm^3^,  median IQR | 2.25  (1.00 – 8.98) | 2.44  (1.02 – 8.75) | 1.75  (0.80 – 8.98) | 0.332 | |
| Scheltens score |  |  |  |  | |
| 0 | 81 (66.9) | 58 (60.4) | 23 (92.0) | 0.010 | |
| 1 | 35 (28.9) | 33 (34.4) | 2 (8.0) |  | |
| 2 or 3 | 5 (4.1) | 5 (5.2) | 0 (0) |  | |
| Fazekas score |  |  |  |  | |
| 0 | 56 (46.3) | 35 (36.5) | 21 (84.0) | <0.001 | |
| 1 | 35 (28.9) | 32 (33.3) | 3 (12.0) |  | |
| 2 or 3 | 30 (24.8) | 29 (30.2) | 1 (4.0) |  | |
| Hemisphere with infarct |  |  |  |  | |
| Dominant hemisphere | 48 (39.7) | 42 (43.8) | 6 (24.0) | 0.139 | |
| Nondominant hemisphere | 71 (58.7) | 52 (54.2) | 19 (76.0) |  | |
| Both | 2 (1.7) | 2 (2.1) | 0 (0) |  | |

Table S3. qEEG characteristics of 121 enrolled participants, stratified by analysis inclusion status

| Variables | Total (n=121) | Analytic inclusion status | | p-value |
| --- | --- | --- | --- | --- |
|  |  | Yes (n=96), n (%) | No (n=25), n (%) |  |
| Frontal DAR | 10.57 (6.07, 15.13) | 10.02 (5.78, 14.92) | 11.58 (7.93, 16.29) | 0.243 |
| Central DAR | 5.41 (2.76, 10.83) | 5.33 (2.53, 10.12) | 5.51 (3.72, 11.64) | 0.611 |
| Temporal DAR | 3.73 (1.89, 7.57) | 3.76 (1.81, 7.52) | 3.70 (2.29, 10.03) | 0.737 |
| Occipital DAR | 1.85 (0.98, 4.37) | 1.83 (0.96, 4.17) | 2.10 (1.00, 5.03) | 0.479 |
| Parietal DAR | 2.55 (1.26, 6.86) | 2.54 (1.18, 6.13) | 3.28 (2.12, 9.61) | 0.095 |
| Global DAR | 4.27 (2.33, 7.87) | 4.26 (2.24, 7.86) | 4.80 (3.16, 8.02) | 0.253 |
| Frontal DFV | 1.59 (1.20, 1.88) | 1.62 (1.22, 1.89) | 1.48 (1.14, 1.88) | 0.375 |
| Central DFV | 1.66 (1.37, 1.98) | 1.71 (1.36, 2.01) | 1.51 (1.38, 1.84) | 0.611 |
| Temporal DFV | 1.68 (1.18, 1.95) | 1.68 (1.21, 1.98) | 1.60 (1.17, 1.83) | 0.464 |
| Occipital DFV | 1.45 (1.19, 1.83) | 1.46 (1.15, 1.82) | 1.45 (1.27, 1.93) | 0.269 |
| Parietal DFV | 1.72 (1.42, 2.04) | 1.68 (1.41, 1.94) | 1.83 (1.53, 2.09) | 0.105 |
| Global DFV | 1.69 (1.31, 1.97) | 1.67 (1.30, 1.99) | 1.71 (1.41, 1.92) | 0.906 |
| Frontal DTABR | 5.62 (2.61, 8.39) | 5.20 (2.59, 8.10) | 7.38 (4.16, 10.54) | 0.063 |
| Central DTABR | 3.56 (1.91, 5.58) | 3.37 (1.75, 5.52) | 4.23 (2.39, 5.58) | 0.444 |
| Temporal DTABR | 2.66 (1.38, 4.87) | 2.64 (1.38, 4.84) | 2.66 (1.38, 6.12) | 0.580 |
| Occipital DTABR | 1.71 (1.03, 3.36) | 1.69 (1.01, 3.04) | 1.75 (1.14, 3.67) | 0.355 |
| Parietal DTABR | 2.60 (1.21, 4.31) | 2.48 (1.06, 4.22) | 2.68 (1.39, 5.95) | 0.093 |
| Global DTABR | 3.13 (1.87, 4.82) | 3.01 (1.80, 4.86) | 3.29 (2.35, 4.62) | 0.199 |
| Frontal PAF | 6.80 (5.70, 8.70) | 6.90 (5.80, 8.83) | 6.10 (5.70, 7.80) | 0.176 |
| Central PAF | 7.80 (6.00, 9.10) | 7.95 (6.00, 9.30) | 7.00 (5.80, 8.60) | 0.306 |
| Temporal PAF | 8.60 (7.40, 9.30) | 8.70 (7.88, 9.40) | 8.30 (6.30, 8.60) | 0.025 |
| Occipital PAF | 9.00 (8.00, 9.50) | 9.00 (8.10, 9.50) | 8.60 (7.70, 9.50) | 0.487 |
| Parietal PAF | 9.10 (8.00, 9.80) | 9.10 (8.07, 9.62) | 9.20 (7.50, 9.90) | 0.543 |
| Global PAF | 8.70 (7.10, 9.50) | 8.75 (7.38, 9.50) | 8.40 (6.30, 9.40) | 0.407 |

Table S4. Comparison of complete-case and multiply-imputed estimates for the final logistic regression model predicting cognitive impairment (MoCA < 26) at 6 months post-stroke.

| **Predictor** | **Complete-case n = 96 OR (95% CI)** | **Multiple imputation n = 119 (96 analytic + 23 imputed), m = 50 OR (95% CI)** |
| --- | --- | --- |
| Occipital DAR | 2.09 (1.45, 3.39) | 2.05 (1.36, 3.08) |
| Frontal PAF | 0.46 (0.27, 0.74) | 0.48 (0.30, 0.79) |
| Education < high school | 7.16 (1.89, 34.18) | 5.24 (1.31, 20.86) |
| AUC (95% CI) | 0.937 (0.890, 0.984) | 0.925 (0.855, 0.962) |

**Sensitivity analysis 2. Outlier exclusion**

Table S5. Participants flagged by all four outlier-detection methods.

| **Patient** | **Manuscript flag** | **qEEG vars \|z\|>3** | **Most extreme variable** | **Mahal-MCD distance** |
| --- | --- | --- | --- | --- |
| Outlier 1 | Yes | 10 of 24 | DAR_t (z = +9.3) | 22,458 |
| (P26) | No | 2 of 24 | DTABr_p (z = +4.9) | 5,072 |
| Outlier 2 | Yes | 5 of 24 | DTABr_p (z = +6.1) | 3,029 |
| (P61) | No | 2 of 24 | DAR_c (z = +6.3) | 1,914 |

Table S6. Occipital DAR effect under five exclusion configurations.

| **Configuration** | **Occipital DAR**  **adjusted OR (95% CI)** | **p-value** | **AUC (95% CI)** |
| --- | --- | --- | --- |
| n = 98 (both outliers retained) | 1.03 (0.95, 1.15) | 0.522 | 0.871 (0.801, 0.941) |
| n = 97 (drop Outlier 2 only) | 1.02 (0.95, 1.15) | 0.567 | 0.868 (0.796, 0.939) |
| n = 97 (drop Outlier 1 only) | 2.09 (1.45, 3.39) | 0.001 | 0.939 (0.892, 0.985) |
| n = 96 (drop both, manuscript) | 2.09 (1.45, 3.39) | 0.001 | 0.937 (0.890, 0.984) |
| n = 94 (drop all 4 flagged) | 2.22 (1.50, 3.70) | <0.001 | 0.943 (0.897, 0.989) |


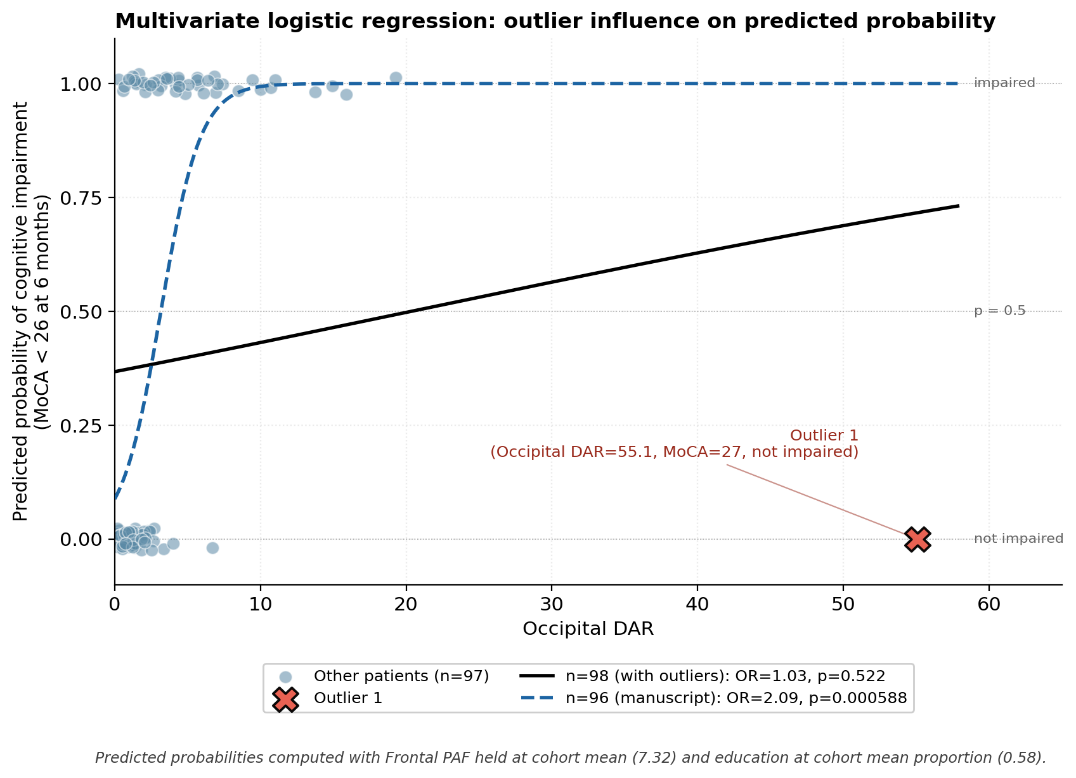


Figure S4. Multivariate logistic regression curves for cognitive impairment (MoCA < 26 at 6 months) on Occipital DAR. The curves are computed from the 3-predictor model {Occipital DAR, Frontal PAF, education < high school} with Frontal PAF held at cohort mean (7.32) and education at cohort mean proportion (0.58). The n = 96 curve (blue dashed) shows a sharp sigmoid (OR = 2.09, p < 0.001); the n = 98 curve (black solid) is nearly flat (OR = 1.03, p = 0.52), having been pulled to accommodate Outlier 1 at Occipital DAR = 55.1 with a not-impaired outcome. Outlier 2 is plotted as a regular point and contributes negligibly to the curve.

**Sensitivity analysis 3. Outcome adjustment and threshold choice**

Table S7. Comparison of predictor estimates under the adjusted (primary) and raw (sensitivity) MoCA outcomes (n = 96).

| **Predictor** | **Adjusted MoCA outcome (primary)** | **Raw MoCA outcome (sensitivity)** |
| --- | --- | --- |
| Occipital DAR | 2.09 (1.45, 3.39) | 1.88 (1.32, 2.96) |
| Frontal PAF | 0.46 (0.27, 0.74) | 0.49 (0.29, 0.75) |
| Education < high school | 7.16 (1.89, 34.18) | 9.67 (2.67, 44.37) |
| AUC (95% CI) | 0.937 (0.889, 0.985) | 0.930 (0.878, 0.981) |

The adjusted MoCA outcome adds 1 point to the raw score for participants with ≤12 years of education; the raw outcome omits this adjustment. Both outcomes use the < 26 impairment cutoff. The model was refit on the same n = 96 analytic sample using the identical model-development procedure (univariable screening followed by forward stepwise logistic regression guided by AIC, with significance-based retention). The same three predictors were retained under both outcomes. OR = odds ratio; AUC = area under the receiver operating characteristic curve.

Table S8. Predictor estimates and discrimination across alternative raw MoCA cutoffs

| **Cutoff** | **Prevalence of cognitive impairment** | **Occipital DAR** | **Frontal PAF** | **Education < high school** | **AUC** |
| --- | --- | --- | --- | --- | --- |
|  |  | ------------Adjusted OR (95% CI)------------- | | |  |
| < 22 | 38/96 (39.6%) | 1.82 (1.34, 2.67) | 0.50 (0.29, 0.79) | 7.03 (1.87, 33.62) | 0.931 |
| < 23 | 42/96 (43.8%) | 1.65 (1.23, 2.38) | 0.50 (0.30, 0.77) | 10.31 (2.85, 47.74) | 0.920 |
| < 24 | 44/96 (45.8%) | 2.09 (1.45, 3.39) | 0.46 (0.27, 0.74) | 7.16 (1.89, 34.18) | 0.937 |
| < 25 | 44/96 (45.8%) | 2.09 (1.45, 3.39) | 0.46 (0.27, 0.74) | 7.16 (1.89, 34.18) | 0.937 |
| < 26 | 47/96 (49.0%) | 1.88 (1.32, 2.96) | 0.49 (0.29, 0.75) | 9.67 (2.67, 44.37) | 0.930 |
| < 27 | 57/96 (59.4%) | 1.91 (1.24, 3.45) | 0.46 (0.26, 0.74) | 21.12 (5.40, 114.48) | 0.947 |

**Sensitivity analysis 4. qEEG measurement timing**

Table S9. qEEG stability across recording days 7–9 (Kruskal–Wallis tests).

| **Variable family** | **Min raw**  **p-value** | **Min Bonferroni**  **p-value** | **Min FDR p-value** | **max ε²** |
| --- | --- | --- | --- | --- |
| DAR (6 channels) | ≥0.10 | 1.00 | 1.00 | <0.02 |
| DTABR (6 channels) | ≥0.13 | 1.00 | 1.00 | <0.02 |
| PAF (6 channels) | ≥0.21 | 1.00 | 1.00 | <0.01 |
| DFV (6 channels) | ≥0.16 | 1.00 | 1.00 | <0.02 |

Table S10. Effect of adding time-from-stroke-to-EEG as a model covariate.

| **Predictor** | **Manuscript Adjusted OR (95% CI)** | **With time-adjusted OR (95% CI)** |
| --- | --- | --- |
| Occipital DAR | 2.09 (1.45, 3.39) | 2.14 (1.46, 3.51) |
| Frontal PAF | 0.46 (0.27, 0.74) | 0.46 (0.26, 0.73) |
| Education < high school | 7.16 (1.89, 34.18) | 7.46 (1.93, 36.94) |
| Time-from-stroke (per day) | - | 1.92 (0.56, 7.81), p = 0.301 |
| AUC | 0.937 | 0.940 |

Table S11**.** Pattern-mixture (δ-adjustment) sensitivity analysis for missing-not-at-random dropout.

| **δ** | **Implied impairment** | **Occipital DAR  OR (95% CI)** | **Frontal PAF  OR (95% CI)** | **Education < HS OR (95% CI)** | **AUC  (95% CI)** |
| --- | --- | --- | --- | --- | --- |
| −2.0 | 18% | 1.93 (1.32-2.82) | 0.48 (0.30-0.80) | 7.29 (1.92-27.58) | 0.92 (0.86-0.96) |
| 0.0 (MAR) | 33% | 2.05 (1.36-3.08) | 0.48 (0.30-0.79) | 5.24 (1.31-20.86) | 0.93 (0.85-0.96) |
| +1.0 | 43% | 2.04 (1.36-3.04) | 0.48 (0.30-0.79) | 4.48 (1.10-18.10) | 0.92 (0.85-0.96) |
| +1.4 (education tipping point) | 47% | 2.00 (1.36-2.95) | 0.48 (0.30-0.78) | 3.90 (1.01-14.93) | 0.92 (0.85-0.96) |
| +2.0 | 53% | 1.96 (1.34-2.86) | 0.47 (0.30-0.77) | 3.38 (0.94-12.15) | 0.92 (0.84-0.96) |
| +5.0 (extreme) | 84% | 1.75 (1.25-2.43) | 0.51 (0.34-0.77) | 2.10 (0.71-6.19) | 0.89 (0.81-0.94) |

Table S12. Worked examples illustrating use of the prediction model: predicted probability of six-month cognitive impairment for three representative patient profiles, computed from the fitted model equation.

| **Patient profile** | **Occipital DAR** | **Frontal PAF** | **Education_low** | **logit(P)** | **P (predicted)** |
| --- | --- | --- | --- | --- | --- |
| Low risk (educated, normal qEEG) | 1.0 | 9.5 | 0 | −4.42 | 0.012 (1.2%) |
| Intermediate (moderate qEEG slowing) | 2.0 | 7.5 | 0 | −2.15 | 0.105 (10.5%) |
| High risk (marked slowing, low edu) | 3.5 | 6.0 | 1 | +2.08 | 0.889 (88.9%) |

*Education_low = 1 if education < high school, 0 otherwise; occipital DAR and frontal PAF are entered on their original continuous scales*

Table S13: Combined-cohort sensitivity analysis: final-model parameters (adjusted OR, 95% CI) in the original first-ever-stroke cohort versus the combined cohort additionally including the 18 recurrent-stroke patients, with and without adjustment for recurrent-stroke status.

| **Predictor** | **Original (n = 96)** | **Combined unadjusted (n = 114)** | **Combined adjusted (n = 114)** |
| --- | --- | --- | --- |
| Occipital DAR | 2.09 (1.45, 3.39) | 1.51 (1.19, 2.02) | 1.48 (1.17, 1.99) |
| Frontal PAF | 0.46 (0.27, 0.74) | 0.43 (0.26, 0.64) | 0.43 (0.27, 0.66) |
| Education < HS | 7.16 (1.89, 34.18) | 5.92 (1.97, 20.08) | 6.05 (2.00, 20.74) |
| Recurrent stroke | - | - | 1.58 (0.36, 8.65) |
| Apparent AUC | 0.937 (0.890, 0.984) | 0.915 (0.859, 0.970) | 0.914 (0.858, 0.970) |

TRIPOD checklist: Prediction Model Development

| **Section/Topic** | **Item** |  | **Checklist Item** | **Page** | |  |
| --- | --- | --- | --- | --- | --- | --- |
| **Title and abstract** | | | | | | |
| Title | 1 | Identify the study as developing and/or validating a multivariable prediction model, the target population, and the outcome to be predicted. | | 1 |  |  |
| Abstract | 2 | Provide a summary of objectives, study design, setting, participants, sample size, predictors, outcome, statistical analysis, results, and conclusions. | | 1 |  |  |
| **Introduction** | | | | | | |
| Background and objectives | 3a | Explain the medical context (including whether diagnostic or prognostic) and rationale for developing or validating the multivariable prediction model, including references to existing models. | | 2-3 | |  |
|  | 3b | Specify the objectives, including whether the study describes the development or validation of the model or both. | | 3 | |  |
| **Methods** | | | | | | |
| Source of data | 4a | Describe the study design or source of data (e.g., randomized trial, cohort, or registry data), separately for the development and validation data sets, if applicable. | | 4 | |  |
|  | 4b | Specify the key study dates, including start of accrual; end of accrual; and, if applicable, end of follow-up. | | 4 | |  |
| Participants | 5a | Specify key elements of the study setting (e.g., primary care, secondary care, general population) including number and location of centres. | | 4 | |  |
|  | 5b | Describe eligibility criteria for participants. | | 4 | |  |
|  | 5c | Give details of treatments received, if relevant. | | x | |  |
| Outcome | 6a | Clearly define the outcome that is predicted by the prediction model, including how and when assessed. | | 9 | |  |
|  | 6b | Report any actions to blind assessment of the outcome to be predicted. | | 9 | |  |
| Predictors | 7a | Clearly define all predictors used in developing or validating the multivariable prediction model, including how and when they were measured. | | 5-8 | |  |
|  | 7b | Report any actions to blind assessment of predictors for the outcome and other predictors. | | 6-7 | |  |
| Sample size | 8 | Explain how the study size was arrived at. | | 4 | |  |
| Missing data | 9 | Describe how missing data were handled (e.g., complete-case analysis, single imputation, multiple imputation) with details of any imputation method. | | 9-10 | |  |
| Statistical analysis methods | 10a | Describe how predictors were handled in the analyses. | | 9 | |  |
|  | 10b | Specify type of model, all model-building procedures (including any predictor selection), and method for internal validation. | | 9-10 | |  |
|  | 10c | For validation, describe how the predictions were calculated. | | 12 | |  |
|  | 10d | Specify all measures used to assess model performance and, if relevant, to compare multiple models. | | 9-10,12 | |  |
|  | 10e | Describe any model updating (e.g., recalibration) arising from the validation, if done. | | 12 | |  |
| Risk groups | 11 | Provide details on how risk groups were created, if done. | | 12 | |  |
| Development vs. validation | 12 | For validation, identify any differences from the development data in setting, eligibility criteria, outcome, and predictors. | | x | |  |
| **Results** | | | | | | |
| Participants | 13a | Describe the flow of participants through the study, including the number of participants with and without the outcome and, if applicable, a summary of the follow-up time. A diagram may be helpful. | | 10 | |  |
|  | 13b | Describe the characteristics of the participants (basic demographics, clinical features, available predictors), including the number of participants with missing data for predictors and outcome. | | 11 | |  |
|  | 13c | For validation, show a comparison with the development data of the distribution of important variables (demographics, predictors and outcome). | | x | |  |
| Model development | 14a | Specify the number of participants and outcome events in each analysis. | | 12 | |  |
|  | 14b | If done, report the unadjusted association between each candidate predictor and outcome. | | 11-12 | |  |
| Model specification | 15a | Present the full prediction model to allow predictions for individuals (i.e., all regression coefficients, and model intercept or baseline survival at a given time point). | | 12 | |  |
|  | 15b | Explain how to use the prediction model. | | 12 | |  |
| Model performance | 16 | Report performance measures (with CIs) for the prediction model. | | 12 | |  |
| Model-updating | 17 | If done, report the results from any model updating (i.e., model specification, model performance). | | 12 | |  |
| **Discussion** | | | | | | |
| Limitations | 18 | Discuss any limitations of the study (such as nonrepresentative sample, few events per predictor, missing data). | | 15-16 | |  |
| Interpretation | 19a | For validation, discuss the results with reference to performance in the development data, and any other validation data. | | 14 | |  |
|  | 19b | Give an overall interpretation of the results, considering objectives, limitations, results from similar studies, and other relevant evidence. | | 13-15 | |  |
| Implications | 20 | Discuss the potential clinical use of the model and implications for future research. | | 15-16 | |  |
| **Other information** | | | | | | |
| Supplementary information | 21 | Provide information about the availability of supplementary resources, such as study protocol, Web calculator, and data sets. | | supplement | |  |
| Funding | 22 | Give the source of funding and the role of the funders for the present study. | | 17 | |  |

STROBE Statement—checklist of items that should be included in reports of observational studies

|  | Item No. | Recommendation | Page  No. |
| --- | --- | --- | --- |
| **Title and abstract** | 1 | (*a*) Indicate the study’s design with a commonly used term in the title or the abstract | 1 |
|  |  | (*b*) Provide in the abstract an informative and balanced summary of what was done and what was found | 1 |
| Introduction | | | |
| Background/rationale | 2 | Explain the scientific background and rationale for the investigation being reported | 2-3 |
| Objectives | 3 | State specific objectives, including any prespecified hypotheses | 3 |
| Methods | | | |
| Study design | 4 | Present key elements of study design early in the paper | 4 |
| Setting | 5 | Describe the setting, locations, and relevant dates, including periods of recruitment, exposure, follow-up, and data collection | 4 |
| Participants | 6 | (*a*) *Cohort study*—Give the eligibility criteria, and the sources and methods of selection of participants. Describe methods of follow-up  *Case-control study*—Give the eligibility criteria, and the sources and methods of case ascertainment and control selection. Give the rationale for the choice of cases and controls  *Cross-sectional study*—Give the eligibility criteria, and the sources and methods of selection of participants | 4 |
|  |  | (*b*) *Cohort study*—For matched studies, give matching criteria and number of exposed and unexposed  *Case-control study*—For matched studies, give matching criteria and the number of controls per case |  |
| Variables | 7 | Clearly define all outcomes, exposures, predictors, potential confounders, and effect modifiers. Give diagnostic criteria, if applicable | 5-9 |
| Data sources/ measurement | 8* | For each variable of interest, give sources of data and details of methods of assessment (measurement). Describe comparability of assessment methods if there is more than one group | *6-8* |
| Bias | 9 | Describe any efforts to address potential sources of bias | 9-10 |
| Study size | 10 | Explain how the study size was arrived at | 4 |

| Quantitative variables | 11 | Explain how quantitative variables were handled in the analyses. If applicable, describe which groupings were chosen and why | 9 |
| --- | --- | --- | --- |
| Statistical methods | 12 | (*a*) Describe all statistical methods, including those used to control for confounding | 9-10 |
|  |  | (*b*) Describe any methods used to examine subgroups and interactions | x |
|  |  | (*c*) Explain how missing data were addressed | 9-10 |
|  |  | (*d*) *Cohort study*—If applicable, explain how loss to follow-up was addressed  *Case-control study*—If applicable, explain how matching of cases and controls was addressed  *Cross-sectional study*—If applicable, describe analytical methods taking account of sampling strategy | 11 |
|  |  | (*e*) Describe any sensitivity analyses | 11 |
| **Results** | | |  |
| Participants | 13* | (a) Report numbers of individuals at each stage of study—eg numbers potentially eligible, examined for eligibility, confirmed eligible, included in the study, completing follow-up, and analysed | 10 |
|  |  | (b) Give reasons for non-participation at each stage | 10 |
|  |  | (c) Consider use of a flow diagram | 10 |
| Descriptive data | 14* | (a) Give characteristics of study participants (eg demographic, clinical, social) and information on exposures and potential confounders | 11 |
|  |  | (b) Indicate number of participants with missing data for each variable of interest | 11 |
|  |  | (c) *Cohort study*—Summarise follow-up time (eg, average and total amount) | 10 |
| Outcome data | 15* | *Cohort study*—Report numbers of outcome events or summary measures over time | *11* |
|  |  | *Case-control study—*Report numbers in each exposure category, or summary measures of exposure |  |
|  |  | *Cross-sectional study—*Report numbers of outcome events or summary measures |  |
| Main results | 16 | (*a*) Give unadjusted estimates and, if applicable, confounder-adjusted estimates and their precision (eg, 95% confidence interval). Make clear which confounders were adjusted for and why they were included | 12 |
|  |  | (*b*) Report category boundaries when continuous variables were categorized |  |
|  |  | (*c*) If relevant, consider translating estimates of relative risk into absolute risk for a meaningful time period |  |

| Other analyses | 17 | Report other analyses done—eg analyses of subgroups and interactions, and sensitivity analyses | 11 |
| --- | --- | --- | --- |
| **Discussion** | | |  |
| Key results | 18 | Summarise key results with reference to study objectives | 13 |
| Limitations | 19 | Discuss limitations of the study, taking into account sources of potential bias or imprecision. Discuss both direction and magnitude of any potential bias | 15-16 |
| Interpretation | 20 | Give a cautious overall interpretation of results considering objectives, limitations, multiplicity of analyses, results from similar studies, and other relevant evidence | 13-15 |
| Generalisability | 21 | Discuss the generalisability (external validity) of the study results | 15-16 |
| **Other information** | | |  |
| Funding | 22 | Give the source of funding and the role of the funders for the present study and, if applicable, for the original study on which the present article is based | 17 |
